# P-1749. Fluconazole disk diffusion on CHROMagar Candida Plus for differentiation between C. auris and C. parapsilosis

**DOI:** 10.1093/ofid/ofaf695.1920

**Published:** 2026-01-11

**Authors:** Sarah Israel, Loujin Ardda, Ariel Israel, Jacob Moran-Gilad, Maya Korem

**Affiliations:** Hadassah Medical Center and the Hebrew University of Jerusalem, Israel, Jerusalem, Yerushalayim, Israel; Hadassah Medical Center and the Hebrew University of Jerusalem, Israel, Jerusalem, Yerushalayim, Israel; Leumit Research Institute, Leumit Health Services, Tel-Aviv, Israel; Department of Epidemiology and Preventive Medicine, School of Public Health, Faculty of Medical & Health Sciences, Tel Aviv University, Tel-Aviv, Israel, Jerusalem, Yerushalayim, Israel; Hadassah Medical Center and the Hebrew University of Jerusalem, Israel, Jerusalem, Yerushalayim, Israel; Hadassah Medical Center and the Hebrew University of Jerusalem, Israel, Jerusalem, Yerushalayim, Israel

## Abstract

**Background:**

*Candida auris* is an emerging, multidrug-resistant yeast requiring strict contact precautions for colonized or infected patients, posing a significant public health threat. Accurate and timely differentiation of *C. auris* from *C. parapsilosis* is crucial, as *C. parapsilosis* typically does not necessitate such precautions and exhibits greater fluconazole susceptibility. However, distinguishing these species on CHROMagar Candida Plus, a common culture medium, can be challenging. This study evaluated fluconazole disk diffusion on CHROMagar Candida Plus as a rapid, potentially resource-sparing method for differentiating *C. auris* from *C. parapsilosis*, particularly in low-resource settings before definitive taxonomic confirmation.

**Methods:**

In vitro testing was performed using clinical isolates of *C. auris* and *C. parapsilosis*. Antifungal susceptibility was assessed via broth microdilution and disk diffusion with varying fluconazole concentrations on both CHROMagar Candida Plus and RPMI agar. Colony color was also recorded.

**Results:**

High categorical agreement between fluconazole disk diffusion on CHROMagar Candida Plus and broth microdilution was observed for both species. A 10 mm zone diameter cutoff on CHROMagar Candida Plus with a 50 µg/ml fluconazole disk yielded 91% sensitivity and 90% specificity in differentiating the two species. A distinct turquoise coloration was noted on the reverse side of *C. auris* colonies on CHROMagar Candida Plus after 48 hours.

**Conclusion:**

Fluconazole disk diffusion on CHROMagar Candida Plus, combined with the observation of a turquoise reverse-side colony coloration, demonstrates promise as a readily implementable and potentially low-resource tool for the accurate and timely presumptive differentiation of *C. auris* and *C. parapsilosis* in clinical laboratories, particularly before definitive taxonomic confirmation.

**Disclosures:**

All Authors: No reported disclosures

